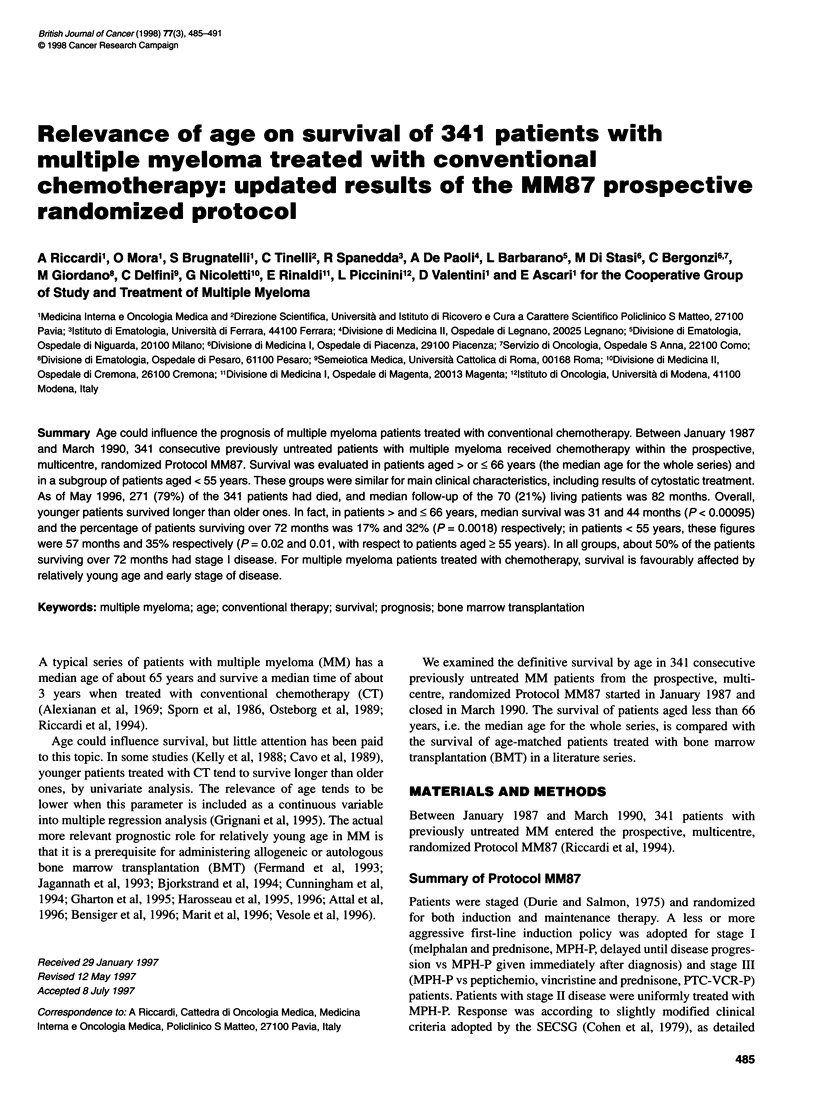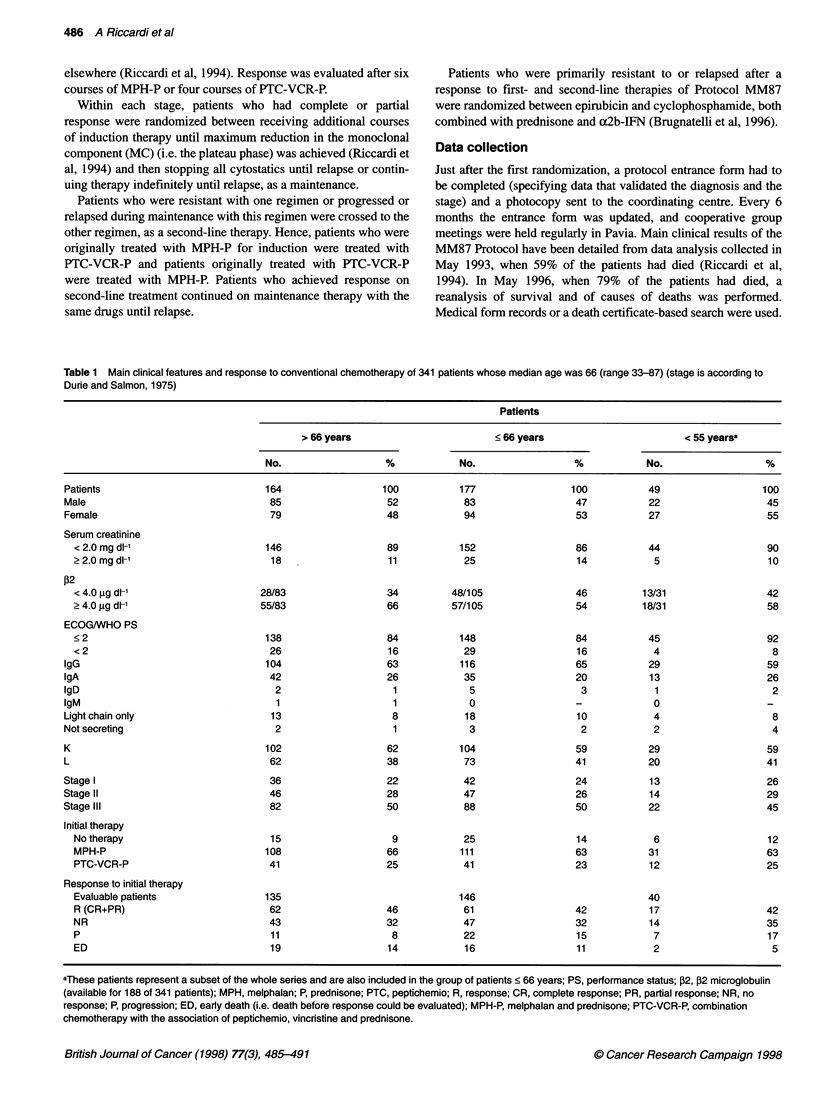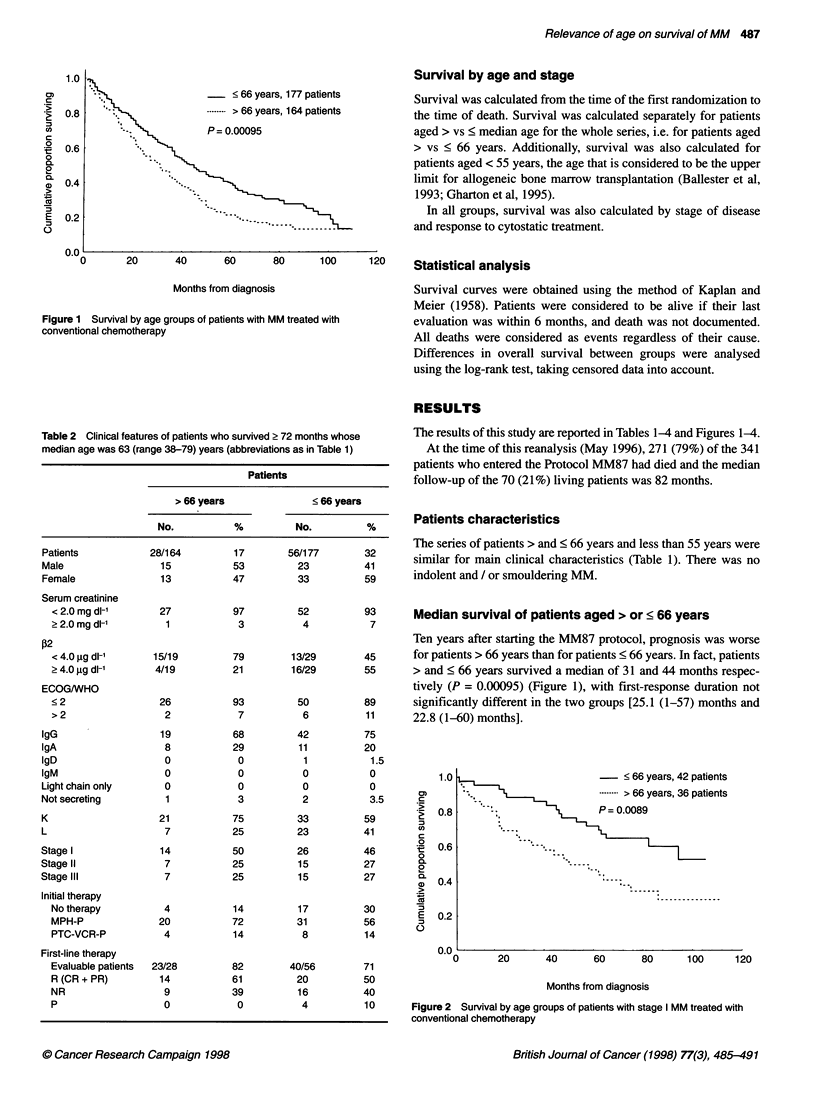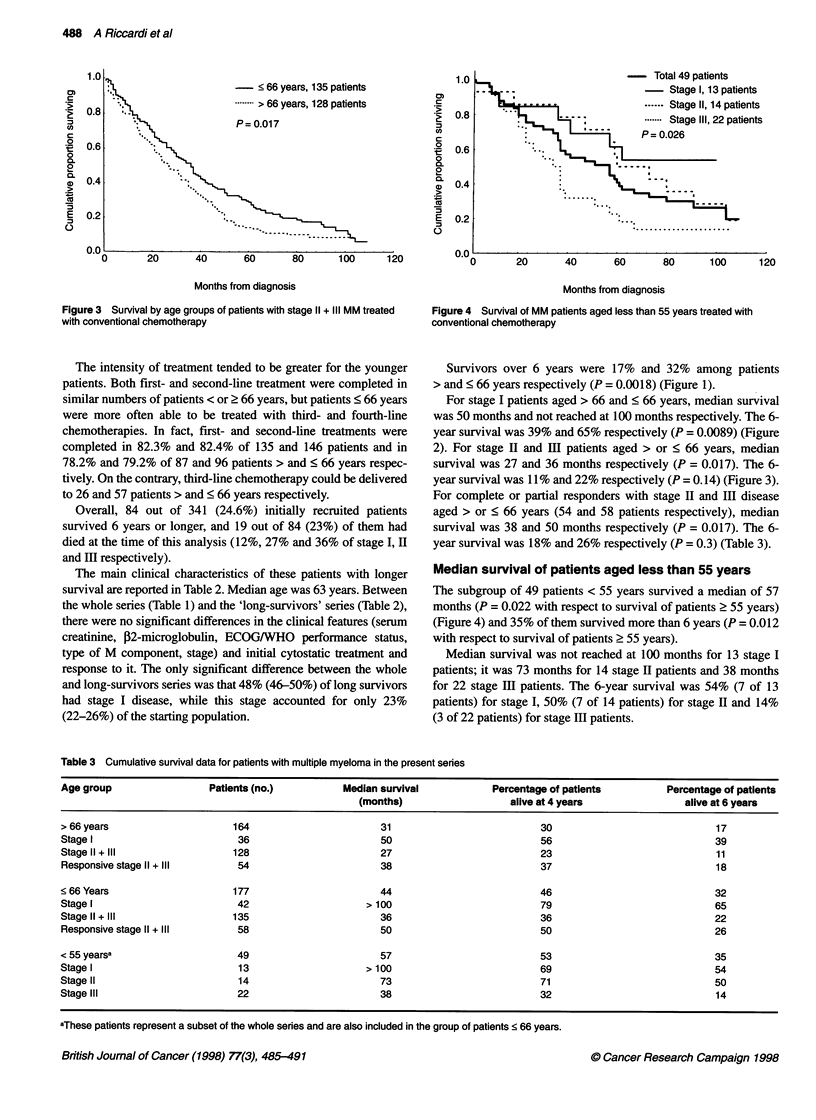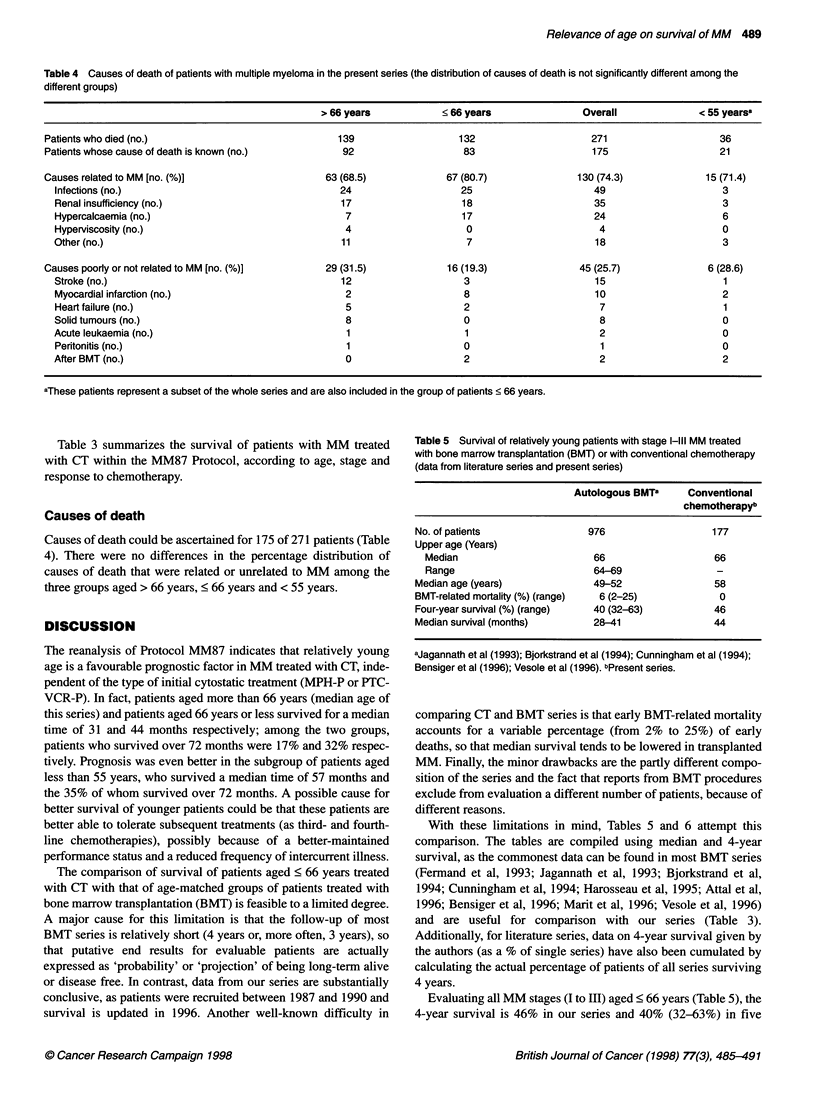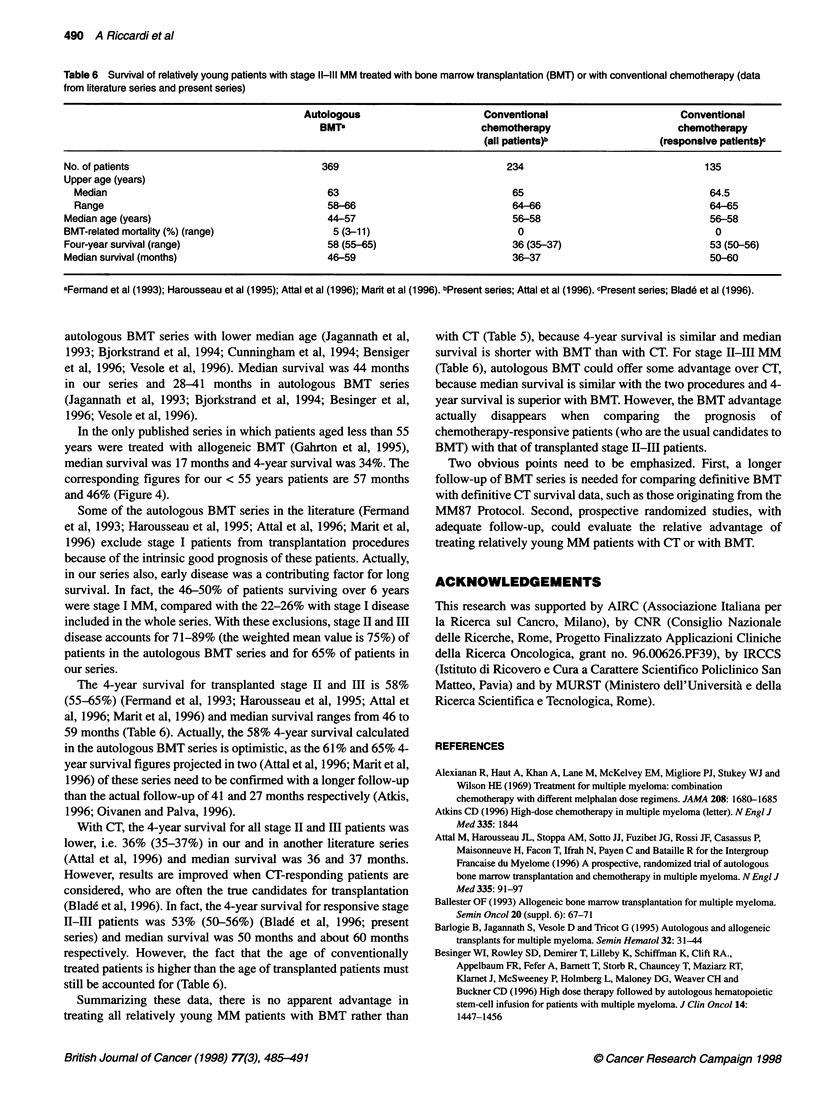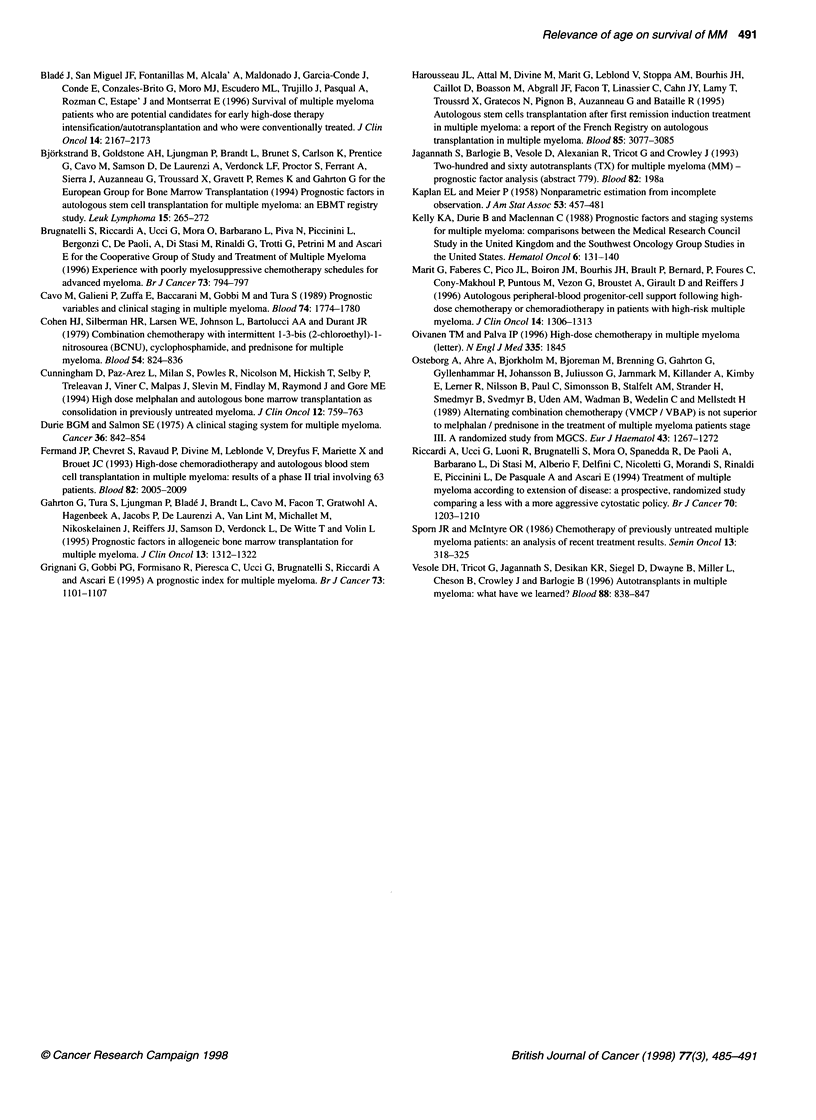# Relevance of age on survival of 341 patients with multiple myeloma treated with conventional chemotherapy: updated results of the MM87 prospective randomized protocol. Cooperative Group of Study and Treatment of Multiple Myeloma.

**DOI:** 10.1038/bjc.1998.77

**Published:** 1998

**Authors:** A. Riccardi, O. Mora, S. Brugnatelli, C. Tinelli, R. Spanedda, A. De Paoli, L. Barbarano, M. Di Stasi, C. Bergonzi, M. Giordano, C. Delfini, G. Nicoletti, E. Rinaldi, L. Piccinini, D. Valentini, E. Ascari

**Affiliations:** Medicina Interna e Oncologia Medica, UniversitÃ and Istituto di Ricovero e Cura a Carattere Scientifico Policlinico S Matteo, Pavia, Italy.

## Abstract

Age could influence the prognosis of multiple myeloma patients treated with conventional chemotherapy. Between January 1987 and March 1990, 341 consecutive previously untreated patients with multiple myeloma received chemotherapy within the prospective, multicentre, randomized Protocol MM87. Survival was evaluated in patients aged > or < or = 66 years (the median age for the whole series) and in a subgroup of patients aged < 55 years. These groups were similar for main clinical characteristics, including results of cytostatic treatment. As of May 1996, 271 (79%) of the 341 patients had died, and median follow-up of the 70 (21%) living patients was 82 months. Overall, younger patients survived longer than older ones. In fact, in patients > and < or = 66 years, median survival was 31 and 44 months (P < 0.00095) and the percentage of patients surviving over 72 months was 17% and 32% (P = 0.0018) respectively; in patients < 55 years, these figures were 57 months and 35% respectively (P = 0.02 and 0.01, with respect to patients aged > 55 years). In all groups, about 50% of the patients surviving over 72 months had stage I disease. For multiple myeloma patients treated with chemotherapy, survival is favourably affected by relatively young age and early stage of disease.


British Journal of Cancer (1998) 77(3), 485–491
© 1998 Cancer Research Campaign

### Relevance of age on survival of 341 patients with multiple myeloma treated with conventional chemotherapy: updated results of the MM87 prospective randomized protocol

**A Riccardi[1], O Mora[1], S Brugnatelli[1], C Tinelli[2], R Spanedda[3], A De Paoli[4], L Barbarano[5], M Di Stasi[6], C Bergonzi[6,7], M Giordano[8], C Delfini[9], G Nicoletti[10], E Rinaldi[11], L Piccinini[12], D Valentini[1] and E Ascari[1] for the Cooperative Group of Study and Treatment of Multiple Myeloma**

[1]Medicina Interna e Oncologia Medica and [2]Direzione Scientifica, Università and Istituto di Ricovero e Cura a Carattere Scientifico Policlinico S Matteo, 27100 Pavia; [3]Istituto di Ematologia, Università di Ferrara, 44100 Ferrara; [4]Divisione di Medicina II, Ospedale di Legnano, 20025 Legnano; [5]Divisione di Ematologia, Ospedale di Niguarda, 20100 Milano; [6]Divisione di Medicina I, Ospedale di Piacenza, 29100 Piacenza; [7]Servizio di Oncologia, Ospedale S Anna, 22100 Como; [8]Divisione di Ematologia, Ospedale di Pesaro, 61100 Pesaro; [9]Semeiotica Medica, Università Cattolica di Roma, 00168 Roma; [10]Divisione di Medicina II, Ospedale di Cremona, 26100 Cremona; [11]Divisione di Medicina I, Ospedale di Magenta, 20013 Magenta; [12]Istituto di Oncologia, Università di Modena, 41100 Modena, Italy

**Summary** Age could influence the prognosis of multiple myeloma patients treated with conventional chemotherapy. Between January 1987 and March 1990, 341 consecutive previously untreated patients with multiple myeloma received chemotherapy within the prospective, multicentre, randomized Protocol MM87. Survival was evaluated in patients aged > or ≤ 66 years (the median age for the whole series) and in a subgroup of patients aged < 55 years. These groups were similar for main clinical characteristics, including results of cytostatic treatment. As of May 1996, 271 (79%) of the 341 patients had died, and median follow-up of the 70 (21%) living patients was 82 months. Overall, younger patients survived longer than older ones. In fact, in patients > and ≤ 66 years, median survival was 31 and 44 months ($P < 0.00095$) and the percentage of patients surviving over 72 months was 17% and 32% ($P = 0.0018$) respectively; in patients < 55 years, these figures were 57 months and 35% respectively ($P = 0.02$ and 0.01, with respect to patients aged ≥ 55 years). In all groups, about 50% of the patients surviving over 72 months had stage I disease. For multiple myeloma patients treated with chemotherapy, survival is favourably affected by relatively young age and early stage of disease.

**Keywords:** multiple myeloma; age; conventional therapy; survival; prognosis; bone marrow transplantation

A typical series of patients with multiple myeloma (MM) has a median age of about 65 years and survive a median time of about 3 years when treated with conventional chemotherapy (CT) (Alexianan et al, 1969; Sporn et al, 1986, Osteborg et al, 1989; Riccardi et al, 1994).

Age could influence survival, but little attention has been paid to this topic. In some studies (Kelly et al, 1988; Cavo et al, 1989), younger patients treated with CT tend to survive longer than older ones, by univariate analysis. The relevance of age tends to be lower when this parameter is included as a continuous variable into multiple regression analysis (Grignani et al, 1995). The actual more relevant prognostic role for relatively young age in MM is that it is a prerequisite for administering allogeneic or autologous bone marrow transplantation (BMT) (Fermand et al, 1993; Jagannath et al, 1993; Bjorkstrand et al, 1994; Cunningham et al, 1994; Gharton et al, 1995; Harosseau et al, 1995, 1996; Attal et al, 1996; Bensiger et al, 1996; Marit et al, 1996; Vesole et al, 1996).

*Received 29 January 1997*
*Revised 12 May 1997*
*Accepted 8 July 1997*

*Correspondence to:* A Riccardi, Cattedra di Oncologia Medica, Medicina Interna e Oncologia Medica, Policlinico S Matteo, 27100 Pavia, Italy

We examined the definitive survival by age in 341 consecutive previously untreated MM patients from the prospective, multicentre, randomized Protocol MM87 started in January 1987 and closed in March 1990. The survival of patients aged less than 66 years, i.e. the median age for the whole series, is compared with the survival of age-matched patients treated with bone marrow transplantation (BMT) in a literature series.

### MATERIALS AND METHODS

Between January 1987 and March 1990, 341 patients with previously untreated MM entered the prospective, multicentre, randomized Protocol MM87 (Riccardi et al, 1994).

### Summary of Protocol MM87

Patients were staged (Durie and Salmon, 1975) and randomized for both induction and maintenance therapy. A less or more aggressive first-line induction policy was adopted for stage I (melphalan and prednisone, MPH-P, delayed until disease progression vs MPH-P given immediately after diagnosis) and stage III (MPH-P vs peptichemio, vincristine and prednisone, PTC-VCR-P) patients. Patients with stage II disease were uniformly treated with MPH-P. Response was according to slightly modified clinical criteria adopted by the SECSG (Cohen et al, 1979), as detailed elsewhere (Riccardi et al, 1994). Response was evaluated after six courses of MPH-P or four courses of PTC-VCR-P.

Within each stage, patients who had complete or partial response were randomized between receiving additional courses of induction therapy until maximum reduction in the monoclonal component (MC) (i.e. the plateau phase) was achieved (Riccardi et al, 1994) and then stopping all cytostatics until relapse or continuing therapy indefinitely until relapse, as a maintenance.

Patients who were resistant with one regimen or progressed or relapsed during maintenance with this regimen were crossed to the other regimen, as a second-line therapy. Hence, patients who were originally treated with MPH-P for induction were treated with PTC-VCR-P and patients originally treated with PTC-VCR-P were treated with MPH-P. Patients who achieved response on second-line treatment continued on maintenance therapy with the same drugs until relapse.

Patients who were primarily resistant to or relapsed after a response to first- and second-line therapies of Protocol MM87 were randomized between epirubicin and cyclophosphamide, both combined with prednisone and α2b-IFN (Brugnatelli et al, 1996).

### Data collection

Just after the first randomization, a protocol entrance form had to be completed (specifying data that validated the diagnosis and the stage) and a photocopy sent to the coordinating centre. Every 6 months the entrance form was updated, and cooperative group meetings were held regularly in Pavia. Main clinical results of the MM87 Protocol have been detailed from data analysis collected in May 1993, when 59% of the patients had died (Riccardi et al, 1994). In May 1996, when 79% of the patients had died, a reanalysis of survival and of causes of deaths was performed. Medical form records or a death certificate-based search were used.

**Table 1** Main clinical features and response to conventional chemotherapy of 341 patients whose median age was 66 (range 33–87) (stage is according to Durie and Salmon, 1975)

| | Patients | | | | | |
|---|---|---|---|---|---|---|
| | > 66 years | | ≤ 66 years | | < 55 years[a] | |
| | No. | % | No. | % | No. | % |
| Patients | 164 | 100 | 177 | 100 | 49 | 100 |
| Male | 85 | 52 | 83 | 47 | 22 | 45 |
| Female | 79 | 48 | 94 | 53 | 27 | 55 |
| Serum creatinine | | | | | | |
| < 2.0 mg dl$^{-1}$ | 146 | 89 | 152 | 86 | 44 | 90 |
| ≥ 2.0 mg dl$^{-1}$ | 18 | 11 | 25 | 14 | 5 | 10 |
| β2 | | | | | | |
| < 4.0 μg dl$^{-1}$ | 28/83 | 34 | 48/105 | 46 | 13/31 | 42 |
| ≥ 4.0 μg dl$^{-1}$ | 55/83 | 66 | 57/105 | 54 | 18/31 | 58 |
| ECOG/WHO PS | | | | | | |
| ≤ 2 | 138 | 84 | 148 | 84 | 45 | 92 |
| < 2 | 26 | 16 | 29 | 16 | 4 | 8 |
| IgG | 104 | 63 | 116 | 65 | 29 | 59 |
| IgA | 42 | 26 | 35 | 20 | 13 | 26 |
| IgD | 2 | 1 | 5 | 3 | 1 | 2 |
| IgM | 1 | 1 | 0 | – | 0 | – |
| Light chain only | 13 | 8 | 18 | 10 | 4 | 8 |
| Not secreting | 2 | 1 | 3 | 2 | 2 | 4 |
| K | 102 | 62 | 104 | 59 | 29 | 59 |
| L | 62 | 38 | 73 | 41 | 20 | 41 |
| Stage I | 36 | 22 | 42 | 24 | 13 | 26 |
| Stage II | 46 | 28 | 47 | 26 | 14 | 29 |
| Stage III | 82 | 50 | 88 | 50 | 22 | 45 |
| Initial therapy | | | | | | |
| No therapy | 15 | 9 | 25 | 14 | 6 | 12 |
| MPH-P | 108 | 66 | 111 | 63 | 31 | 63 |
| PTC-VCR-P | 41 | 25 | 41 | 23 | 12 | 25 |
| Response to initial therapy | | | | | | |
| Evaluable patients | 135 | | 146 | | 40 | |
| R (CR+PR) | 62 | 46 | 61 | 42 | 17 | 42 |
| NR | 43 | 32 | 47 | 32 | 14 | 35 |
| P | 11 | 8 | 22 | 15 | 7 | 17 |
| ED | 19 | 14 | 16 | 11 | 2 | 5 |

[a]These patients represent a subset of the whole series and are also included in the group of patients ≤ 66 years; PS, performance status; β2, β2 microglobulin (available for 188 of 341 patients); MPH, melphalan; P, prednisone; PTC, peptichemio; R, response; CR, complete response; PR, partial response; NR, no response; P, progression; ED, early death (i.e. death before response could be evaluated); MPH-P, melphalan and prednisone; PTC-VCR-P, combination chemotherapy with the association of peptichemio, vincristine and prednisone.

© Cancer Research Campaign 1998

**Figure 1** Survival by age groups of patients with MM treated with conventional chemotherapy

**Table 2** Clinical features of patients who survived ≥ 72 months whose median age was 63 (range 38–79) years (abbreviations as in Table 1)

| | Patients | | | |
|---|---|---|---|---|
| | > 66 years | | ≤ 66 years | |
| | No. | % | No. | % |
| Patients | 28/164 | 17 | 56/177 | 32 |
| Male | 15 | 53 | 23 | 41 |
| Female | 13 | 47 | 33 | 59 |
| Serum creatinine | | | | |
| < 2.0 mg dl$^{-1}$ | 27 | 97 | 52 | 93 |
| ≥ 2.0 mg dl$^{-1}$ | 1 | 3 | 4 | 7 |
| β2 | | | | |
| < 4.0 μg dl$^{-1}$ | 15/19 | 79 | 13/29 | 45 |
| ≥ 4.0 μg dl$^{-1}$ | 4/19 | 21 | 16/29 | 55 |
| ECOG/WHO | | | | |
| ≤ 2 | 26 | 93 | 50 | 89 |
| > 2 | 2 | 7 | 6 | 11 |
| IgG | 19 | 68 | 42 | 75 |
| IgA | 8 | 29 | 11 | 20 |
| IgD | 0 | 0 | 1 | 1.5 |
| IgM | 0 | 0 | 0 | 0 |
| Light chain only | 0 | 0 | 0 | 0 |
| Not secreting | 1 | 3 | 2 | 3.5 |
| K | 21 | 75 | 33 | 59 |
| L | 7 | 25 | 23 | 41 |
| Stage I | 14 | 50 | 26 | 46 |
| Stage II | 7 | 25 | 15 | 27 |
| Stage III | 7 | 25 | 15 | 27 |
| Initial therapy | | | | |
| No therapy | 4 | 14 | 17 | 30 |
| MPH-P | 20 | 72 | 31 | 56 |
| PTC-VCR-P | 4 | 14 | 8 | 14 |
| First-line therapy | | | | |
| Evaluable patients | 23/28 | 82 | 40/56 | 71 |
| R (CR + PR) | 14 | 61 | 20 | 50 |
| NR | 9 | 39 | 16 | 40 |
| P | 0 | 0 | 4 | 10 |

### Survival by age and stage

Survival was calculated from the time of the first randomization to the time of death. Survival was calculated separately for patients aged > vs ≤ median age for the whole series, i.e. for patients aged > vs ≤ 66 years. Additionally, survival was also calculated for patients aged < 55 years, the age that is considered to be the upper limit for allogeneic bone marrow transplantation (Ballester et al, 1993; Gharton et al, 1995).

In all groups, survival was also calculated by stage of disease and response to cytostatic treatment.

### Statistical analysis

Survival curves were obtained using the method of Kaplan and Meier (1958). Patients were considered to be alive if their last evaluation was within 6 months, and death was not documented. All deaths were considered as events regardless of their cause. Differences in overall survival between groups were analysed using the log-rank test, taking censored data into account.

### RESULTS

The results of this study are reported in Tables 1–4 and Figures 1–4.

At the time of this reanalysis (May 1996), 271 (79%) of the 341 patients who entered the Protocol MM87 had died and the median follow-up of the 70 (21%) living patients was 82 months.

### Patients characteristics

The series of patients > and ≤ 66 years and less than 55 years were similar for main clinical characteristics (Table 1). There was no indolent and / or smouldering MM.

### Median survival of patients aged > or ≤ 66 years

Ten years after starting the MM87 protocol, prognosis was worse for patients > 66 years than for patients ≤ 66 years. In fact, patients > and ≤ 66 years survived a median of 31 and 44 months respectively (*P* = 0.00095) (Figure 1), with first-response duration not significantly different in the two groups [25.1 (1–57) months and 22.8 (1–60) months].

**Figure 2** Survival by age groups of patients with stage I MM treated with conventional chemotherapy

**Figure 3** Survival by age groups of patients with stage II + III MM treated with conventional chemotherapy

**Figure 4** Survival of MM patients aged less than 55 years treated with conventional chemotherapy

The intensity of treatment tended to be greater for the younger patients. Both first- and second-line treatment were completed in similar numbers of patients < or ≥ 66 years, but patients ≤ 66 years were more often able to be treated with third- and fourth-line chemotherapies. In fact, first- and second-line treatments were completed in 82.3% and 82.4% of 135 and 146 patients and in 78.2% and 79.2% of 87 and 96 patients > and ≤ 66 years respectively. On the contrary, third-line chemotherapy could be delivered to 26 and 57 patients > and ≤ 66 years respectively.

Overall, 84 out of 341 (24.6%) initially recruited patients survived 6 years or longer, and 19 out of 84 (23%) of them had died at the time of this analysis (12%, 27% and 36% of stage I, II and III respectively).

The main clinical characteristics of these patients with longer survival are reported in Table 2. Median age was 63 years. Between the whole series (Table 1) and the 'long-survivors' series (Table 2), there were no significant differences in the clinical features (serum creatinine, β2-microglobulin, ECOG/WHO performance status, type of M component, stage) and initial cytostatic treatment and response to it. The only significant difference between the whole and long-survivors series was that 48% (46–50%) of long survivors had stage I disease, while this stage accounted for only 23% (22–26%) of the starting population.

Survivors over 6 years were 17% and 32% among patients > and ≤ 66 years respectively ($P = 0.0018$) (Figure 1).

For stage I patients aged > 66 and ≤ 66 years, median survival was 50 months and not reached at 100 months respectively. The 6-year survival was 39% and 65% respectively ($P = 0.0089$) (Figure 2). For stage II and III patients aged > or ≤ 66 years, median survival was 27 and 36 months respectively ($P = 0.017$). The 6-year survival was 11% and 22% respectively ($P = 0.14$) (Figure 3). For complete or partial responders with stage II and III disease aged > or ≤ 66 years (54 and 58 patients respectively), median survival was 38 and 50 months respectively ($P = 0.017$). The 6-year survival was 18% and 26% respectively ($P = 0.3$) (Table 3).

### Median survival of patients aged less than 55 years

The subgroup of 49 patients < 55 years survived a median of 57 months ($P = 0.022$ with respect to survival of patients ≥ 55 years) (Figure 4) and 35% of them survived more than 6 years ($P = 0.012$ with respect to survival of patients ≥ 55 years).

Median survival was not reached at 100 months for 13 stage I patients; it was 73 months for 14 stage II patients and 38 months for 22 stage III patients. The 6-year survival was 54% (7 of 13 patients) for stage I, 50% (7 of 14 patients) for stage II and 14% (3 of 22 patients) for stage III patients.

**Table 3** Cumulative survival data for patients with multiple myeloma in the present series

| Age group | Patients (no.) | Median survival (months) | Percentage of patients alive at 4 years | Percentage of patients alive at 6 years |
|---|---|---|---|---|
| > 66 years | 164 | 31 | 30 | 17 |
| Stage I | 36 | 50 | 56 | 39 |
| Stage II + III | 128 | 27 | 23 | 11 |
| Responsive stage II + III | 54 | 38 | 37 | 18 |
| ≤ 66 Years | 177 | 44 | 46 | 32 |
| Stage I | 42 | > 100 | 79 | 65 |
| Stage II + III | 135 | 36 | 36 | 22 |
| Responsive stage II + III | 58 | 50 | 50 | 26 |
| < 55 years[a] | 49 | 57 | 53 | 35 |
| Stage I | 13 | > 100 | 69 | 54 |
| Stage II | 14 | 73 | 71 | 50 |
| Stage III | 22 | 38 | 32 | 14 |

[a]These patients represent a subset of the whole series and are also included in the group of patients ≤ 66 years.

 © Cancer Research Campaign 1998

**Table 4** Causes of death of patients with multiple myeloma in the present series (the distribution of causes of death is not significantly different among the different groups)

| | > 66 years | ≤ 66 years | Overall | < 55 years[a] |
|---|---|---|---|---|
| Patients who died (no.) | 139 | 132 | 271 | 36 |
| Patients whose cause of death is known (no.) | 92 | 83 | 175 | 21 |
| Causes related to MM [no. (%)] | 63 (68.5) | 67 (80.7) | 130 (74.3) | 15 (71.4) |
| Infections (no.) | 24 | 25 | 49 | 3 |
| Renal insufficiency (no.) | 17 | 18 | 35 | 3 |
| Hypercalcaemia (no.) | 7 | 17 | 24 | 6 |
| Hyperviscosity (no.) | 4 | 0 | 4 | 0 |
| Other (no.) | 11 | 7 | 18 | 3 |
| Causes poorly or not related to MM [no. (%)] | 29 (31.5) | 16 (19.3) | 45 (25.7) | 6 (28.6) |
| Stroke (no.) | 12 | 3 | 15 | 1 |
| Myocardial infarction (no.) | 2 | 8 | 10 | 2 |
| Heart failure (no.) | 5 | 2 | 7 | 1 |
| Solid tumours (no.) | 8 | 0 | 8 | 0 |
| Acute leukaemia (no.) | 1 | 1 | 2 | 0 |
| Peritonitis (no.) | 1 | 0 | 1 | 0 |
| After BMT (no.) | 0 | 2 | 2 | 2 |

[a]These patients represent a subset of the whole series and are also included in the group of patients ≤ 66 years.

Table 3 summarizes the survival of patients with MM treated with CT within the MM87 Protocol, according to age, stage and response to chemotherapy.

### Causes of death

Causes of death could be ascertained for 175 of 271 patients (Table 4). There were no differences in the percentage distribution of causes of death that were related or unrelated to MM among the three groups aged > 66 years, ≤ 66 years and < 55 years.

### DISCUSSION

The reanalysis of Protocol MM87 indicates that relatively young age is a favourable prognostic factor in MM treated with CT, independent of the type of initial cytostatic treatment (MPH-P or PTC-VCR-P). In fact, patients aged more than 66 years (median age of this series) and patients aged 66 years or less survived for a median time of 31 and 44 months respectively; among the two groups, patients who survived over 72 months were 17% and 32% respectively. Prognosis was even better in the subgroup of patients aged less than 55 years, who survived a median time of 57 months and the 35% of whom survived over 72 months. A possible cause for better survival of younger patients could be that these patients are better able to tolerate subsequent treatments (as third- and fourth-line chemotherapies), possibly because of a better-maintained performance status and a reduced frequency of intercurrent illness.

The comparison of survival of patients aged ≤ 66 years treated with CT with that of age-matched groups of patients treated with bone marrow transplantation (BMT) is feasible to a limited degree. A major cause for this limitation is that the follow-up of most BMT series is relatively short (4 years or, more often, 3 years), so that putative end results for evaluable patients are actually expressed as ‘probability’ or ‘projection’ of being long-term alive or disease free. In contrast, data from our series are substantially conclusive, as patients were recruited between 1987 and 1990 and survival is updated in 1996. Another well-known difficulty in comparing CT and BMT series is that early BMT-related mortality accounts for a variable percentage (from 2% to 25%) of early deaths, so that median survival tends to be lowered in transplanted MM. Finally, the minor drawbacks are the partly different composition of the series and the fact that reports from BMT procedures exclude from evaluation a different number of patients, because of different reasons.

**Table 5** Survival of relatively young patients with stage I–III MM treated with bone marrow transplantation (BMT) or with conventional chemotherapy (data from literature series and present series)

| | Autologous BMT[a] | Conventional chemotherapy[b] |
|---|---|---|
| No. of patients | 976 | 177 |
| Upper age (Years) | | |
| Median | 66 | 66 |
| Range | 64–69 | – |
| Median age (years) | 49–52 | 58 |
| BMT-related mortality (%) (range) | 6 (2–25) | 0 |
| Four-year survival (%) (range) | 40 (32–63) | 46 |
| Median survival (months) | 28–41 | 44 |

[a]Jagannath et al (1993); Bjorkstrand et al (1994); Cunningham et al (1994); Bensiger et al (1996); Vesole et al (1996). [b]Present series.

With these limitations in mind, Tables 5 and 6 attempt this comparison. The tables are compiled using median and 4-year survival, as the commonest data can be found in most BMT series (Fermand et al, 1993; Jagannath et al, 1993; Bjorkstrand et al, 1994; Cunningham et al, 1994; Harosseau et al, 1995; Attal et al, 1996; Bensiger et al, 1996; Marit et al, 1996; Vesole et al, 1996) and are useful for comparison with our series (Table 3). Additionally, for literature series, data on 4-year survival given by the authors (as a % of single series) have also been cumulated by calculating the actual percentage of patients of all series surviving 4 years.

Evaluating all MM stages (I to III) aged ≤ 66 years (Table 5), the 4-year survival is 46% in our series and 40% (32–63%) in five

© Cancer Research Campaign 1998

**Table 6** Survival of relatively young patients with stage II–III MM treated with bone marrow transplantation (BMT) or with conventional chemotherapy (data from literature series and present series)

| | Autologous BMT[a] | Conventional chemotherapy (all patients)[b] | Conventional chemotherapy (responsive patients)[c] |
|---|---|---|---|
| No. of patients | 369 | 234 | 135 |
| Upper age (years) | | | |
| Median | 63 | 65 | 64.5 |
| Range | 58–66 | 64–66 | 64–65 |
| Median age (years) | 44–57 | 56–58 | 56–58 |
| BMT-related mortality (%) (range) | 5 (3–11) | 0 | 0 |
| Four-year survival (range) | 58 (55–65) | 36 (35–37) | 53 (50–56) |
| Median survival (months) | 46–59 | 36–37 | 50–60 |

[a]Fermand et al (1993); Harousseau et al (1995); Attal et al (1996); Marit et al (1996). [b]Present series; Attal et al (1996). [c]Present series; Bladé et al (1996).

autologous BMT series with lower median age (Jagannath et al, 1993; Bjorkstrand et al, 1994; Cunningham et al, 1994; Bensiger et al, 1996; Vesole et al, 1996). Median survival was 44 months in our series and 28–41 months in autologous BMT series (Jagannath et al, 1993; Bjorkstrand et al, 1994; Besinger et al, 1996; Vesole et al, 1996).

In the only published series in which patients aged less than 55 years were treated with allogeneic BMT (Gahrton et al, 1995), median survival was 17 months and 4-year survival was 34%. The corresponding figures for our < 55 years patients are 57 months and 46% (Figure 4).

Some of the autologous BMT series in the literature (Fermand et al, 1993; Harousseau et al, 1995; Attal et al, 1996; Marit et al, 1996) exclude stage I patients from transplantation procedures because of the intrinsic good prognosis of these patients. Actually, in our series also, early disease was a contributing factor for long survival. In fact, the 46–50% of patients surviving over 6 years were stage I MM, compared with the 22–26% with stage I disease included in the whole series. With these exclusions, stage II and III disease accounts for 71–89% (the weighted mean value is 75%) of patients in the autologous BMT series and for 65% of patients in our series.

The 4-year survival for transplanted stage II and III is 58% (55–65%) (Fermand et al, 1993; Harousseau et al, 1995; Attal et al, 1996; Marit et al, 1996) and median survival ranges from 46 to 59 months (Table 6). Actually, the 58% 4-year survival calculated in the autologous BMT series is optimistic, as the 61% and 65% 4-year survival figures projected in two (Attal et al, 1996; Marit et al, 1996) of these series need to be confirmed with a longer follow-up than the actual follow-up of 41 and 27 months respectively (Atkis, 1996; Oivanen and Palva, 1996).

With CT, the 4-year survival for all stage II and III patients was lower, i.e. 36% (35–37%) in our and in another literature series (Attal et al, 1996) and median survival was 36 and 37 months. However, results are improved when CT-responding patients are considered, who are often the true candidates for transplantation (Bladé et al, 1996). In fact, the 4-year survival for responsive stage II–III patients was 53% (50–56%) (Bladé et al, 1996; present series) and median survival was 50 months and about 60 months respectively. However, the fact that the age of conventionally treated patients is higher than the age of transplanted patients must still be accounted for (Table 6).

Summarizing these data, there is no apparent advantage in treating all relatively young MM patients with BMT rather than with CT (Table 5), because 4-year survival is similar and median survival is shorter with BMT than with CT. For stage II–III MM (Table 6), autologous BMT could offer some advantage over CT, because median survival is similar with the two procedures and 4-year survival is superior with BMT. However, the BMT advantage actually disappears when comparing the prognosis of chemotherapy-responsive patients (who are the usual candidates to BMT) with that of transplanted stage II–III patients.

Two obvious points need to be emphasized. First, a longer follow-up of BMT series is needed for comparing definitive BMT with definitive CT survival data, such as those originating from the MM87 Protocol. Second, prospective randomized studies, with adequate follow-up, could evaluate the relative advantage of treating relatively young MM patients with CT or with BMT.

### ACKNOWLEDGEMENTS

This research was supported by AIRC (Associazione Italiana per la Ricerca sul Cancro, Milano), by CNR (Consiglio Nazionale delle Ricerche, Rome, Progetto Finalizzato Applicazioni Cliniche della Ricerca Oncologica, grant no. 96.00626.PF39), by IRCCS (Istituto di Ricovero e Cura a Carattere Scientifico Policlinico San Matteo, Pavia) and by MURST (Ministero dell'Università e della Ricerca Scientifica e Tecnologica, Rome).

### REFERENCES

Alexianan R, Haut A, Khan A, Lane M, McKelvey EM, Migliore PJ, Stukey WJ and Wilson HE (1969) Treatment for multiple myeloma: combination chemotherapy with different melphalan dose regimens. *JAMA* **208**: 1680–1685

Atkins CD (1996) High-dose chemotherapy in multiple myeloma (letter). *N Engl J Med* **335**: 1844

Attal M, Harousseau JL, Stoppa AM, Sotto JJ, Fuzibet JG, Rossi JF, Casassus P, Maisonneuve H, Facon T, Ifrah N, Payen C and Bataille R for the Intergroup Francaise du Myelome (1996) A prospective, randomized trial of autologous bone marrow transplantation and chemotherapy in multiple myeloma. *N Engl J Med* **335**: 91–97

Ballester OF (1993) Allogeneic bone marrow transplantation for multiple myeloma. *Semin Oncol* **20** (suppl. 6): 67–71

Barlogie B, Jagannath S, Vesole D and Tricot G (1995) Autologous and allogeneic transplants for multiple myeloma. *Semin Hematol* **32**: 31–44

Besinger WI, Rowley SD, Demirer T, Lilleby K, Schiffman K, Clift RA., Appelbaum FR, Fefer A, Barnett T, Storb R, Chauncey T, Maziarz RT, Klarnet J, McSweeney P, Holmberg L, Maloney DG, Weaver CH and Buckner CD (1996) High dose therapy followed by autologous hematopoietic stem-cell infusion for patients with multiple myeloma. *J Clin Oncol* **14**: 1447–1456

© Cancer Research Campaign 1998

Bladé J, San Miguel JF, Fontanillas M, Alcala' A, Maldonado J, Garcia-Conde J, Conde E, Conzales-Brito G, Moro MJ, Escudero ML, Trujillo J, Pasqual A, Rozman C, Estape' J and Montserrat E (1996) Survival of multiple myeloma patients who are potential candidates for early high-dose therapy intensification/autotransplantation and who were conventionally treated. *J Clin Oncol* **14**: 2167–2173

Björkstrand B, Goldstone AH, Ljungman P, Brandt L, Brunet S, Carlson K, Prentice G, Cavo M, Samson D, De Laurenzi A, Verdonck LF, Proctor S, Ferrant A, Sierra J, Auzanneau G, Troussard X, Gravett P, Remes K and Gahrton G for the European Group for Bone Marrow Transplantation (1994) Prognostic factors in autologous stem cell transplantation for multiple myeloma: an EBMT registry study. *Leuk Lymphoma* **15**: 265–272

Brugnatelli S, Riccardi A, Ucci G, Mora O, Barbarano L, Piva N, Piccinini L, Bergonzi C, De Paoli, A, Di Stasi M, Rinaldi G, Trotti G, Petrini M and Ascari E for the Cooperative Group of Study and Treatment of Multiple Myeloma (1996) Experience with poorly myelosuppressive chemotherapy schedules for advanced myeloma. *Br J Cancer* **73**: 794–797

Cavo M, Galieni P, Zuffa E, Baccarani M, Gobbi M and Tura S (1989) Prognostic variables and clinical staging in multiple myeloma. *Blood* **74**: 1774–1780

Cohen HJ, Silberman HR, Larsen WE, Johnson L, Bartolucci AA and Durant JR (1979) Combination chemotherapy with intermittent 1-3-bis (2-chloroethyl)-1-nitrosourea (BCNU), cyclophosphamide, and prednisone for multiple myeloma. *Blood* **54**: 824–836

Cunningham D, Paz-Arez L, Milan S, Powles R, Nicolson M, Hickish T, Selby P, Treleavan J, Viner C, Malpas J, Slevin M, Findlay M, Raymond J and Gore ME (1994) High dose melphalan and autologous bone marrow transplantation as consolidation in previously untreated myeloma. *J Clin Oncol* **12**: 759–763

Durie BGM and Salmon SE (1975) A clinical staging system for multiple myeloma. *Cancer* **36**: 842–854

Fermand JP, Chevret S, Ravaud P, Divine M, Leblonde V, Dreyfus F, Mariette X and Brouet JC (1993) High-dose chemoradiotherapy and autologous blood stem cell transplantation in multiple myeloma: results of a phase II trial involving 63 patients. *Blood* **82**: 2005–2009

Gahrton G, Tura S, Ljungman P, Bladé J, Brandt L, Cavo M, Facon T, Gratwohl A, Hagenbeek A, Jacobs P, De Laurenzi A, Van Lint M, Michallet M, Nikoskelainen J, Reiffers JJ, Samson D, Verdonck L, De Witte T and Volin L (1995) Prognostic factors in allogeneic bone marrow transplantation for multiple myeloma. *J Clin Oncol* **13**: 1312–1322

Grignani G, Gobbi PG, Formisano R, Pieresca C, Ucci G, Brugnatelli S, Riccardi A and Ascari E (1995) A prognostic index for multiple myeloma. *Br J Cancer* **73**: 1101–1107

Harousseau JL, Attal M, Divine M, Marit G, Leblond V, Stoppa AM, Bourhis JH, Caillot D, Boasson M, Abgrall JF, Facon T, Linassier C, Cahn JY, Lamy T, Troussrd X, Gratecos N, Pignon B, Auzanneau G and Bataille R (1995) Autologous stem cells transplantation after first remission induction treatment in multiple myeloma: a report of the French Registry on autologous transplantation in multiple myeloma. *Blood* **85**: 3077–3085

Jagannath S, Barlogie B, Vesole D, Alexanian R, Tricot G and Crowley J (1993) Two-hundred and sixty autotransplants (TX) for multiple myeloma (MM) – prognostic factor analysis (abstract 779). *Blood* **82**: 198a

Kaplan EL and Meier P (1958) Nonparametric estimation from incomplete observation. *J Am Stat Assoc* **53**: 457–481

Kelly KA, Durie B and Maclennan C (1988) Prognostic factors and staging systems for multiple myeloma: comparisons between the Medical Research Council Study in the United Kingdom and the Southwest Oncology Group Studies in the United States. *Hematol Oncol* **6**: 131–140

Marit G, Faberes C, Pico JL, Boiron JM, Bourhis JH, Brault P, Bernard, P, Foures C, Cony-Makhoul P, Puntous M, Vezon G, Broustet A, Girault D and Reiffers J (1996) Autologous peripheral-blood progenitor-cell support following high-dose chemotherapy or chemoradiotherapy in patients with high-risk multiple myeloma. *J Clin Oncol* **14**: 1306–1313

Oivanen TM and Palva IP (1996) High-dose chemotherapy in multiple myeloma (letter). *N Engl J Med* **335**: 1845

Osteborg A, Ahre A, Bjorkholm M, Bjoreman M, Brenning G, Gahrton G, Gyllenhammar H, Johansson B, Juliusson G, Jarnmark M, Killander A, Kimby E, Lerner R, Nilsson B, Paul C, Simonsson B, Stalfelt AM, Strander H, Smedmyr B, Svedmyr B, Uden AM, Wadman B, Wedelin C and Mellstedt H (1989) Alternating combination chemotherapy (VMCP / VBAP) is not superior to melphalan / prednisone in the treatment of multiple myeloma patients stage III. A randomized study from MGCS. *Eur J Haematol* **43**: 1267–1272

Riccardi A, Ucci G, Luoni R, Brugnatelli S, Mora O, Spanedda R, De Paoli A, Barbarano L, Di Stasi M, Alberio F, Delfini C, Nicoletti G, Morandi S, Rinaldi E, Piccinini L, De Pasquale A and Ascari E (1994) Treatment of multiple myeloma according to extension of disease: a prospective, randomized study comparing a less with a more aggressive cytostatic policy. *Br J Cancer* **70**: 1203–1210

Sporn JR and McIntyre OR (1986) Chemotherapy of previously untreated multiple myeloma patients: an analysis of recent treatment results. *Semin Oncol* **13**: 318–325

Vesole DH, Tricot G, Jagannath S, Desikan KR, Siegel D, Dwayne B, Miller L, Cheson B, Crowley J and Barlogie B (1996) Autotransplants in multiple myeloma: what have we learned? *Blood* **88**: 838–847

© Cancer Research Campaign 1998